# The Cell Cycle Timing of Human Papillomavirus DNA Replication

**DOI:** 10.1371/journal.pone.0131675

**Published:** 2015-07-01

**Authors:** Tormi Reinson, Liisi Henno, Mart Toots, Mart Ustav, Mart Ustav

**Affiliations:** 1 University of Tartu, Institute of Technology Department of Biomedical Technology, Nooruse 1, 50411, Tartu, Estonia; 2 Estonian Academy of Sciences, Kohtu 6, 10130, Tallinn, Estonia; 3 Icosagen Cell Factory OÜ, Nooruse 9, 50411, Tartu, Estonia; National Institute of Health - National Cancer Institute, UNITED STATES

## Abstract

Viruses manipulate the cell cycle of the host cell to optimize conditions for more efficient viral genome replication. One strategy utilized by DNA viruses is to replicate their genomes non-concurrently with the host genome; in this case, the viral genome is amplified outside S phase. This phenomenon has also been described for human papillomavirus (HPV) vegetative genome replication, which occurs in G2-arrested cells; however, the precise timing of viral DNA replication during initial and stable replication phases has not been studied. We developed a new method to quantitate newly synthesized DNA levels and used this method in combination with cell cycle synchronization to show that viral DNA replication is initiated during S phase and is extended to G2 during initial amplification but follows the replication pattern of cellular DNA during S phase in the stable maintenance phase. E1 and E2 protein overexpression changes the replication time from S only to both the S and G2 phases in cells that stably maintain viral episomes. These data demonstrate that the active synthesis and replication of the HPV genome are extended into the G2 phase to amplify its copy number and the duration of HPV genome replication is controlled by the level of the viral replication proteins E1 and E2. Using the G2 phase for genome amplification may be an important adaptation that allows exploitation of changing cellular conditions during cell cycle progression. We also describe a new method to quantify newly synthesized viral DNA levels and discuss its benefits for HPV research.

## Introduction

Human papilloma virus (HPV) infects basal keratinocytes of the stratified epithelium, and its life cycle is tightly linked to the normal differentiation process of the epidermis. HPV DNA replication during its life cycle occurs in three separate phases (reviewed in [1, 2]). After viral entry into the cell nucleus and the activation of viral gene expression, the viral genome copy number increases to several hundred copies per cell during the initial phase of genome amplification. This phase is followed by a stable maintenance phase in which the viral genome copy number is kept constant during cell divisions. The final phase of HPV life cycle is the vegetative amplification when a second increase in the viral genome copy number occurs.

E1 and E2 are the only two viral proteins that are directly involved in papillomavirus (PV) genome replication [3]. E1 is the viral DNA helicase, which uses energy from ATP hydrolysis to unwind dsDNA during replication (reviewed in [4]). E2 is a transcription and segregation factor, and its role in PV DNA replication is to direct E1 to the viral replication origin by increasing the E1 origin-binding specificity [5]. After the initial binding and melting of the dsDNA at the origin, E1 forms two hexameric complexes on the DNA, each encircling one of the opposite DNA strands [6]. These two E1 hexamers recruit cellular replication factors for the bidirectional synthesis of viral DNA. This E1-based replication mechanism relies on the same cellular proteins that are used for host DNA replication during S phase. However, increasing evidence has suggested that HPV can also use recombination-dependent replication (RDR) to synthesize viral DNA [7, 8]. RDR is used by dsDNA viruses for ori-independent assembly of the replisome on viral DNA as a result of replication fork stalling [9]. The activation of the DNA-damage response components ATR [10] and ATM [11, 12] in viral DNA replication centers also indicates that RDR might be involved in HPV DNA replication. However, the involvement of DNA damage response (DDR) pathways varies during different viral replication phases. While vegetative amplification is dependent on DNA-damage response activation, stable maintenance is independent of DDR, as shown by the different requirements for the DDR proteins ATM [12] and Nbs1 [13] during these phases.

Many dsDNA viruses affect the cell cycle of infected host cells. For example, herpes viruses, which have large genomes that encode most of the necessary replication proteins, arrest the cell cycle in G1/G0 phase during lytic infection (reviewed in [14]), which helps the virus avoid competition for DNA-synthesis resources such as nucleotide pools for the extensive replication of its own genome. However, during latent infection, herpes viruses use an S phase-based replication strategy where only cellular replication proteins are used for replicating viral genomes. In contrast, various viruses, including small dsDNA viruses, have been shown to cause G2/M cell cycle arrest [1]. The large T antigen of JC polyomavirus causes cells to arrest in G2/M, and this arrest is necessary for the effective replication of the viral genome [15]. During vegetative amplification, papillomaviruses arrest the cell cycle in G2 through the action of the E7 protein [16]. These G2-arrested cells are also the sites of extensive viral DNA replication during vegetative amplification [17]. We demonstrated previously that the initial amplification of HPV can also occur during G2 because a considerable amount of cells containing viral replication centers are also positive for the G2 marker cyclin B1 [10]. However, no cell cycle arrest has been detected; no change in the cell cycle profile has been observed during the initial amplification of HPV genomes.

Although small DNA viruses can replicate their genomes during G2, how or why these viruses do so remains unclear. HPV genome replication seems to occur in G2 if the genome is extensively amplified, as in case of vegetative amplification or the intense transient replication of the HPV18/E8ˉ mutant. However, the timing of DNA replication for wt HPV during the initial amplification and stable maintenance phases has not been studied. The present study used the synchronization of the cell cycle in combination with the quantification of newly synthesized DNA to show that stable replication occurs only in S phase, while initial amplification starts in S and continues in G2.

## Materials and Methods

### Cell lines and transfection

The U2OS cell line (obtained from American Type Culture Collection; ATCC no: HTB-96), which was used in all experiments, was grown in Iscove’s modified Dulbecco’s medium (IMDM) supplemented with 10% fetal bovine serum. The electroporation of viral genomes was performed as described previously [3], except that no carrier DNA was added to the transfections. A Bio-Rad Gene Pulser XCell II apparatus (Bio-Rad Laboratories) equipped with a capacitance extender at 220 V and a capacitance of 975 μF was used in all experiments.

U2OS cells were cotransfected with the HPV18 genome and the selection vector pBabePuro [18] for the generation of HPV18 stable cell pools. Selection with puromycin was started at 72 hours after transfection and continued for 3 days to eliminate untransfected cells. Then, antibiotic-free medium was used until the cells reached confluency and during subsequent normal passaging. HPV genome amplification was induced as described previously [19]. Briefly, HPV genome-maintaining cells were seeded at a density of 1 million cells per 100 mm plate and grown without splitting for the indicated times. The media was changed every two days.

A peptide-based transfection reagent (Reagent 007, Icosagen, Estonia) was used for the transfection of E1 and E2 expression constructs during cell cycle synchronization. The manufacturer’s protocol was used with slight modifications as follows: 1 μg of DNA and 7.6 μl of the transfection reagent were diluted in a final volume of 50 μl, and DNA-peptide complexes were formed by incubating the mixture at room temperature for 45 minutes. Then, complexes corresponding to 250 ng of the expression construct were added to a 60 mm plate containing 2 ml of medium and incubated for 12 hours.

### Plasmids

The plasmids pMHE1-18 and pQMNE2-18 were used for HPV18 E1 and E2 protein expression, respectively; these plasmids have been described previously [20]. HPV genomes that were used for transfection were produced in minicircle form by inducing the recombination and degradation of vector DNA from viral genomes [10, 21]. The HPV18/E8ˉ genome was constructed by introducing a point mutation in the start codon of the E8 gene (AUG-ACG). pFRG-EGFP plasmid (Icosagen, Estonia), which expresses EGFP under the CMV promoter was used in the transfection efficiency experient.

### DNA extraction

Cells were washed twice with PBS and lysed in lysis solution (20 mM Tris-HCl (pH 8.0), 100 mM NaCl, 10 mM EDTA, and 0.2% SDS) for total genomic DNA extraction. The cell lysates were treated overnight with proteinase K (0.2 μg/ml), extracted with phenol-chloroform and precipitated with 2 volumes of 96% ethanol. DNA pellets were dissolved in TE buffer containing 20 μg/ml RNase A and incubated for 1 hour at 37°C. The DNA was precipitated with 96% ethanol, washed with 70% ethanol, and dissolved in TE. DNA concentrations were measured using a NanoDrop Spectrophotometer ND1000.

### Quantitative PCR (qPCR)

**Fig 1:**
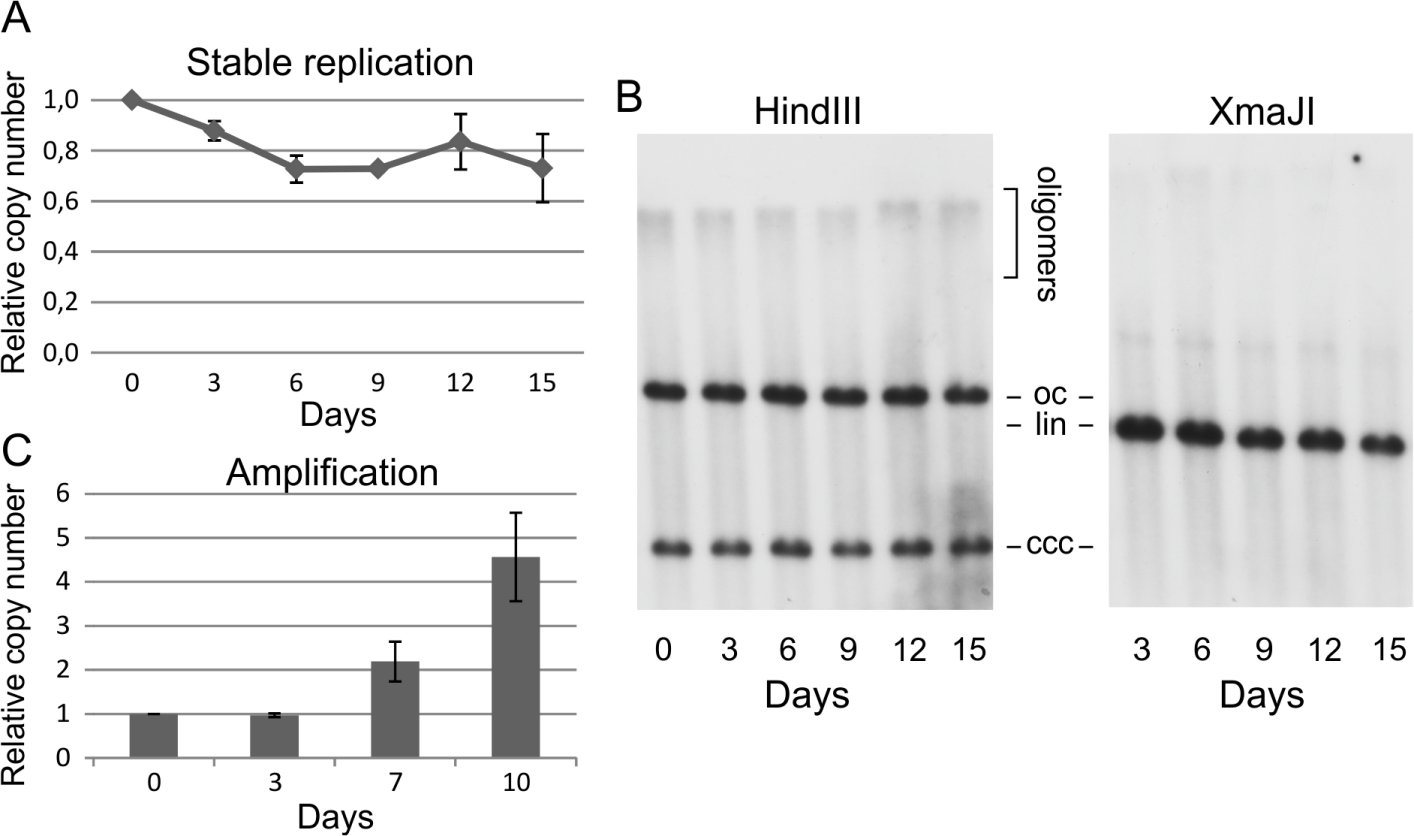
Stable replication of the HPV18 genome in the U2OS cell line.

Equal amounts of total DNA were digested with DpnI to remove the input DNA and with an HPV18 genome-linearizing enzyme. The reaction mix was diluted, and 1 ng of total DNA was used in a single PCR, along with 300 nM forward and reverse primers, 2 μl of commercial master mix and 5x HOT FIREPol EvaGreen qPCR mix (Solis BioDyne) in a 10 μl total reaction volume. Amplification was performed on a 7900HT Real-Time PCR System (Applied Biosystems). The comparative threshold cycle (ΔΔC_t_) method was used for the HPV genome quantification shown in Fig 1 by comparing HPV genome-specific signals to the signals of a reference (ribosomal DNA: rDNA) in the cellular genome. The change in the threshold cycle value (ΔC_t_) of the sequence of interest (HPV, rDNA, and alpha-amylase (Amy)) was used to quantitate the relative amounts of newly synthesized DNA. The following primers were used: HPV18, GTGCATCCCAGCAGTAAG and AAACCAGCCGTTACAACC; rDNA, GCGGCGTTATTCCCATGACC and GGAATTGACGGAAGGGCACC; Amy, ACTCAAGGTAAGTAACAGCCCACGG and CTACACGTGGCTTGGTCACTTCATG.

### Southern blot analysis

Two micrograms of total DNA was digested with an appropriate enzyme for linearization and with DpnI, resolved on an agarose gel, blotted, and hybridized with an HPV18 genome sequence-specific probe labeled with [alpha-32P]dCTP using random priming (DecaLabel Kit; Thermo-Scientific). Signals specific to the HPV genome were detected with autoradiographic exposure using X-ray film (Fuji).

### Quantification of newly synthesized DNA

Newly synthesized DNA was pulse labeled with 10 μM 5-Ethynyl-2'-deoxyuridine (EdU) for one hour before cell lysis. Then, total DNA was extracted and sonicated to generate approximately 500–1000 bp fragments. A click reaction was performed for 30 minutes at room temperature using a Click-iT Cell Reaction Buffer Kit (Invitrogen) in a 50 μl total volume containing 1 μg of DNA, 1x reaction buffer, 5 μl of buffer additive, 2 mM CuSO_4_ and 20 μM biotin-azide. The reaction volume was increased to 500 μl with TE, and DNA was precipitated with 200 mM NaCl, 20 μg of glycogen and two volumes of 96% ethanol. The DNA precipitate was washed with 70% ethanol and dissolved in 20 μl of TE buffer. The DNA concentration was measured using a NanoDrop spectrophotometer, and equal amounts of biotinylated DNA were used in each binding reaction, along with 10 μl of M-270 streptavidin Dynabeads (Invitrogen). The reaction was performed in binding and wash buffer (BW buffer, 10 mM Tris-HCl (pH 7.5), 1 M NaCl, 1 mM EDTA and 0.1% Tween 20) with gentle rocking for 1 hour at room temperature. The beads were washed three times with BW buffer and twice with TE. Then, the beads were resuspended in 200 μl of TE, and 5 μl of suspension was directly used in a qPCR reaction for quantifying the viral and cellular DNA.

The click reaction for quantifying total EdU^+^ DNA was performed with slight modifications as follows: Eighty nanograms total DNA was used in a 20-μl total reaction volume in the presence of 200 μM biotin-azide, 1x reaction buffer, 2 μl of buffer additive and 2 mM CuSO_4_. The reaction was performed for one hour at room temperature, directly followed by transferring the DNA to a nylon transfer membrane (Membrane Solutions LLC) using a dot blot apparatus (Convertible Filtration Manifold System, Gibco BRL) in 10x SSC (1.5 M NaCl and 150 mM sodium citrate). The DNA was crosslinked to the filter using a UV Stratalinker 1800 apparatus (Stratagene), blocked for 1 hour with 5% BSA in BW buffer (500 mM NaCl was used instead of 1 M in all steps of this protocol), incubated for one hour in the same buffer with 25 ng/ml Pierce high sensitivity streptavidin HRP conjugate (Thermo Scientific) and washed once for 5 minutes and 3 times for 15 minutes with BW buffer. Chemiluminescent detection was performed with Amersham ECL Western Blotting Detection Reagent (GE Healthcare)**.** An ImageQuant RT ECL imager (GE Healthcare) was used for dot blot intensity quantification.

### Cell cycle analysis and synchronization

Cells were incubated in the presence of 5 μg/ml aphidicolin for 14 hours, washed three times with PBS and then grown for another 12 hours in 100 ng/ml nocodazole to synchronize the cell cycle into early mitosis. The cells were released into fresh media after being washed with PBS three times, and several time points were taken during the next 20 hours. DNA-peptide complexes were added to the medium together with nocodazole for 12 hours to transfect cells with the E1 and E2 expression constructs. Cell cycle analysis was performed as described previously [10].

Immunofluorescence analysis (IF)

IF was performed exactly as described previously [10]. Briefly, cells were fixed with 4% paraformaldehyde, and permeabilized with 0.5% Triton X-100 in PBS. The cells were then blocked with 5% bovine serum albumin (BSA) and incubated consecutively with primary and secondary antibodies in antibody binding solution (3% BSA in PBS). The cells were placed on glass slides using a mounting medium containing 0.1 mM 4,6-diamidino-2-phenylindole (DAPI) and examined using a confocal microscope (Carl Zeiss LSM 710).

## Results

### Stable replication of the HPV18 genome in U2OS cell line

We demonstrated previously that the U2OS cell line is suitable for studying different phases of HPV genome replication [19, 22]. We studied the amplification of the HPV18 genome by transfecting U2OS cells with viral genomes using electroporation [7, 10, 19, 22]. Cell pools stably maintaining the viral genome were created to analyze the HPV stable maintenance stage by cotransfecting the viral genome with the selection marker plasmid, followed by subsequent puromycin selection. The puromycin-selected cell pools were followed for 15 days to assess the stable maintenance of the HPV genome in these cells. Cell samples were lysed for total DNA extraction every three days (Fig 1), and the viral genome replication signal was measured by either qPCR (Fig 1A) or Southern blotting (Fig 1B). The HPV genome was shown to be stably maintained in these cell pools. A minimal (approximately 20%) decrease in the viral genome copy number was detected by qPCR during the initial period (Fig 1A). Southern blot analysis was performed on samples digested with either an enzyme that does not cut the HPV18 genome (noncutter, HindIII) or an enzyme that linearizes the viral genome (XmaJI) to obtain information regarding the molecular forms of the viral genome in these cells. The HPV genome signal appeared at heights corresponding to monomeric covalently closed circular (ccc), open circular (oc) and oligomeric forms, in the case of noncutter digestion. However, the HPV signal appeared as one band equivalent to a linear monomer after treatment with a linearizing enzyme. These data indicate that the HPV18 genome is stably maintained as an extrachromosomal episome for at least 15 days in cell pools that were created from the U2OS cell line.

Cell lines that stably maintained HPV18 episomes were followed for 15 days. The relative copy number of the viral genome was determined by qPCR (A). The average of two experiments is shown in A, with error bars representing standard deviations. Total DNA samples were cut with either an HPV18 noncutting (HindIII) or a linearizing (XmaJI) enzyme for Southern blot analyses (B). (C) The relative copy number of the HPV18 genome during the induction of amplification under dense culture conditions.

The final phase of the HPV replication cycle is vegetative amplification; during this phase, the viral genome is actively replicated, and its genome copy number increases multiple times. We demonstrated previously that this amplification also occurs in U2OS cell lines that stably maintain HPV episomes when these cell lines are grown under dense culture conditions [19]. Next, the ability of stably maintained viral genomes in the previously described cell pools to begin vegetative amplification was tested. Cells were seeded at fixed subconfluent densities and grown for 10 days without splitting but with media exchange every two days (Fig 1C). The viral genome copy number was measured in cells immediately before seeding and at different time points during this 10-day growth period. The viral genome copy number increased 4–5 times by the tenth day, therefore imitating the start of vegetative amplification and indicating that the HPV genome in U2OS cell pools does indeed stay active.

### Quantification of newly synthesized DNA

We were next interested in comparing the timing of viral DNA replication in transient and stable replication systems. We have previously used immunofluorescence (IF) to study HPV18/E8ˉ transient DNA replication timing and shown that replication foci of this viral mutant can be detected in cyclin B1 positive cells, indicating that its replication can take place during the G2 phase of the cell cycle [10]. However, HPV18/E8ˉ does not express the repressor protein E8/E2 and therefore has remarkably higher transient replication levels compared to the HPV18 wt genome. Lower replication levels make it difficult to detect transient replication centers of wt HPV18 with IF. Morever, IF cannot be used to study the stable DNA replication timing of the HPV18 wt genome because viral replication foci cannot be detected in this case. To address this problem, we developed a new click chemistry-based technique to quantify newly synthesized DNA for use in combination with cell cycle synchronization.

**Fig 2:**
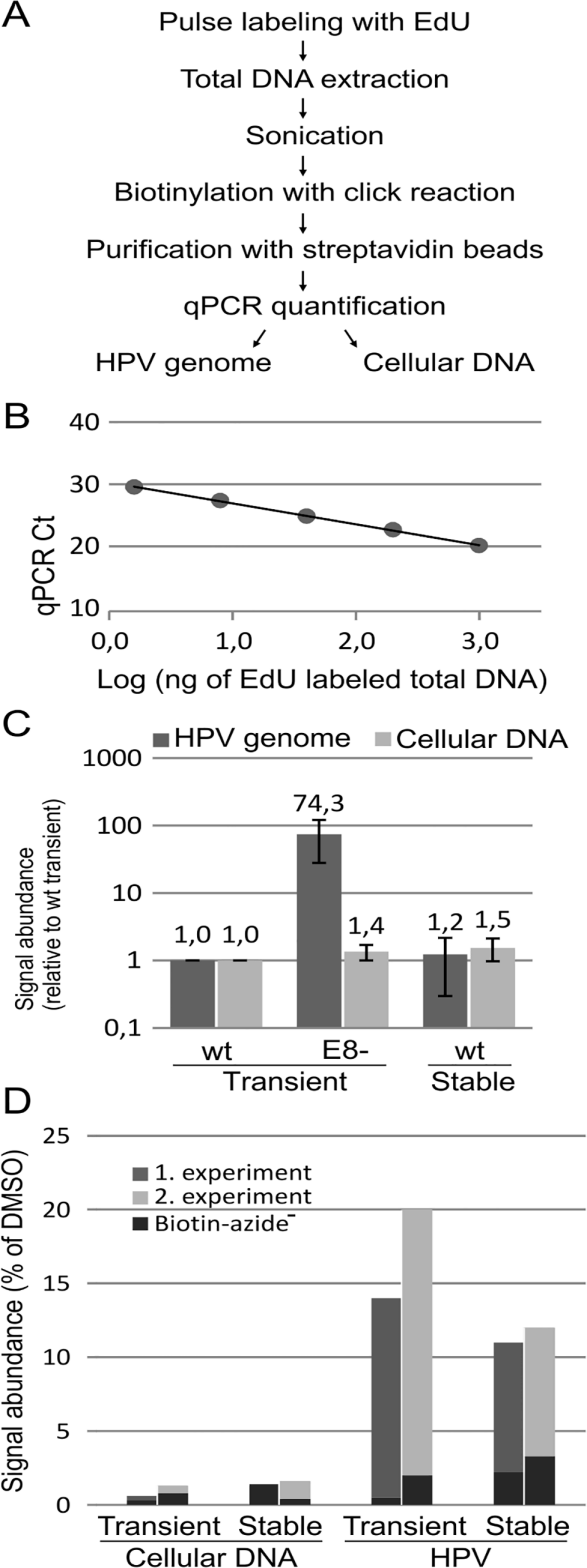
Quantification of newly synthesized DNA.

In this assay, newly synthesized DNA is pulse labeled with the nucleoside analog EdU for one hour before total DNA extraction and DNA sonication into approximately 1 kb fragments (Fig 2A). Then, the click reaction between the incorporated EdU and biotin-azide is performed to biotinylate the newly synthesized DNA for subsequent purification with streptavidin beads. Then, the precipitated DNA is quantified by qPCR using primers that are specific to either viral or cellular sequences. Dilutions of HPV-positive (U2OS cells transiently transfected with HPV18/E8ˉ) total DNA were prepared using DNA extracted from HPV-negative U2OS cells to determine the linear detection range of the newly synthesized viral DNA (Fig 2B). This assay has at least a three-log linear range.

Assay scheme for the click reaction-based quantification of newly synthesized DNA (A, see text for details). Dilutions of EdU-labeled total DNA, which was extracted from HPV18/E8ˉ transfected cells, were prepared to determine the linear detection range of the assay (B). Quantification of newly synthesized viral and cellular DNA in transient and stable HPV18 replication systems (C-D). U2OS cells were transfected with either wt or E8ˉ HPV18 genomes for transient replication analysis, and cell lines that stably maintained the wt HPV18 genome were used for the stable replication assay. Primers against HPV or the cellular genome (ribosomal DNA: rDNA) were used in the qPCR for the quantification of either viral or host DNA replication. The average of three experiments is shown in C, with error bars representing standard deviations. The effect of aphidicolin on the abundance of newly synthesized DNA is shown in D. Aphidicolin (5 μg/ml) or 0.5% DMSO as the vehicle control was added to the media 3 hours before cell lysis (2 hours before the start of the EdU pulse). The results from two separate experiments are shown as a percentage of the aphidicolin-treated sample to the respective DMSO control sample. Mock controls without biotin-azide addition to the click reaction are shown as black bars.

Next, we measured the replication intensities of viral and cellular DNA in transient and stable HPV-replication systems (Fig 2C). As expected, the abundance of newly synthesized cellular DNA was identical in the case of U2OS cells stably transfected with HPV18 wt or transiently transfected with either the HPV18 wt or E8ˉ genome. HPV18 wt transient and stable replication intensities were also approximately identical. This result might seem somewhat counterintuitive initially because the viral genome copy number per cell increases during initial amplification, whereas this value remains constant during stable maintenance. However, this assay does not measure how many times the viral genome is multiplied; instead, this assay measures the incorporation of the nucleosides into the DNA, i.e., the rate of DNA synthesis, which depends on the total number of active replication forks. In contrast, knocking out the E8/E2 repressor protein increased the transient replication intensity of HPV18 by approximately 100 fold. All these data are consistent with previous knowledge regarding HPV replication, indicating that this assay is suitable for quantifying newly synthesized HPV genomic DNA levels.

The levels of newly synthesized DNA were measured in the presence of aphidicolin, which is a known DNA polymerase alpha inhibitor that stops cellular DNA replication and therefore arrests the cell cycle in S phase to further evaluate this assay (Fig 2D). Both cellular and viral DNA replication activity was measured in transient and stable HPV replication systems. The signal from cells that were preincubated with 5 μg/ml aphidicolin for two hours before the start of the EdU pulse was compared to that of the DMSO vehicle control. Aphidicolin treatment decreased the signal of newly synthesized cellular DNA by approximately 100 fold, similar to the background levels of biotin-azide-negative click reactions. HPV replication was also extremely sensitive to aphidicolin treatment; HPV replication decreased by 80–85% compared to the DMSO control in the case of transient HPV18 replication and by approximately 90% in case of stable replication. Newly synthesized HPV genome signals remained considerably higher compared with those of biotin-azide-negative mock controls, indicating that some fraction of HPV replication is not sensitive to aphidicolin treatment.

### Cell cycle timing of HPV18 DNA replication

**Fig 3:**
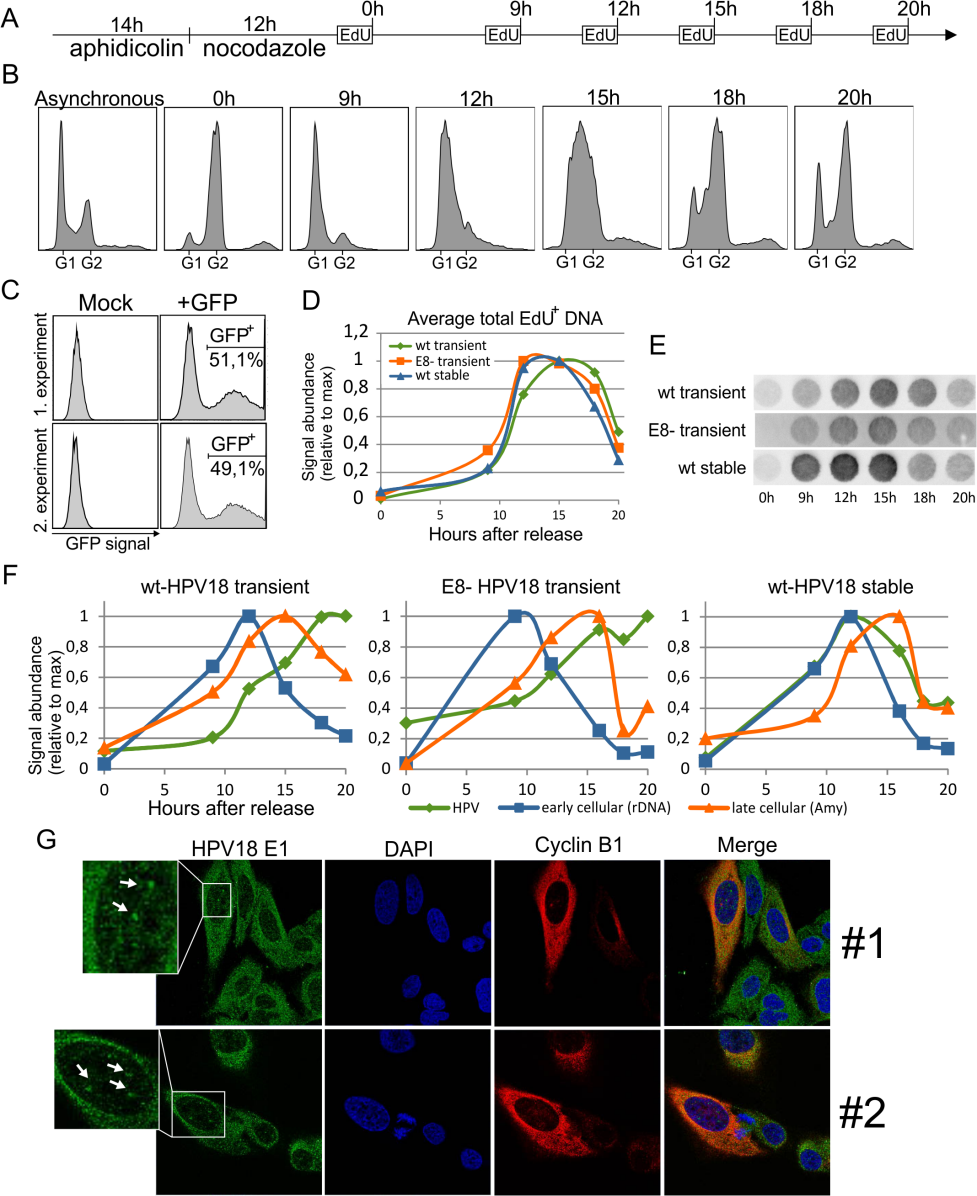
Difference in cell cycle timing of HPV18 DNA replication during transient and stable replication phases.

Next, we combined this new assay with cell cycle synchronization to analyze the cell cycle timing of HPV18 wt DNA replication during the initial amplification and stable maintenance phases (Fig 3). Initial amplification of HPV18/E8ˉ genome was also studied for compariosn with our previous findings [10]. U2OS cells were transfected with either HPV18 wt or E8ˉ genomes, and synchronization was started at three days post-transfection to study the initial amplification phase. Transfection efficiency of the electroporation protocol was measured with GFP expression plasmid (Fig 3C) and it turned out that considerable part of the cells, around 50%, are transfected under used conditions. The cell pool stably maintaining the HPV18 wt genome was used for experiments concerning the stable maintenance phase. Cell populations were synchronized to mitosis by successive incubations with aphidicolin and nocodazole (Fig 3A). Then, the cells were released into fresh media, and several time points were taken during the next 20 hours, which allowed cells to progress through G1 and S phases and to reach G2 phase at the final time points (Fig 3B). A one-hour EdU pulse was performed before each time point to measure the DNA synthesis rates of viral and cellular genomes during different cell cycle phases (Fig 3D, 3E and 3F). Overall, the DNA synthesis activity was first measured by quantifying the total levels of EdU-positive DNA at different time points (Fig 3D and 3E). This quantification was performed by biotinylating EdU-positive DNA via a click reaction; then, this biotinylated DNA was transferred to a nylon membrane and incubated with streptavidin-HRP for detection via an enhanced chemiluminescent reaction. The fraction of EdU-positive DNA began to increase starting at 9 hours after release from the nocodazole block, reached its maximum between 12–15 hours, and then decreased at later time points.

Cell cycle synchronization, together with newly synthesized DNA quantification, was used to measure HPV DNA replication activity in different cell cycle phases (assay scheme in A). The cell cycle was synchronized by sequential aphidicolin and nocodazole treatments, followed by release in fresh media and the collection of time points over the next 20 hours. A 1-hour EdU pulse was performed before the cells were lysed for total DNA extraction. Cell cycle profiles for each time point were determined by propidium iodide staining and by flow cytometry (B). Transfection of a GFP expression vector was used to measure the efficiency of transfection (C). DNA replication timing was measured in U2OS cells transiently transfected with wt or E8ˉ HPV18 genomes or with cells stably maintaining episomes of the HPV18 genome (D-F). Total EdU^+^ DNA was quantified by blotting the DNA onto nylon transfer membrane, incubating the membrane with streptavidin HRP conjugate, and detecting by ECL (D, E). The average of three different synchronization experiments is shown in D, and single examples are shown in E. The quantification of newly synthesized DNA is shown in F. Primers against the viral genome, early replicating (rDNA) and late-replicating (Amy) host DNA were used for qPCR. The signals indicating newly synthesized DNA abundance were normalized to the maximum value of the series and plotted against time after release from the nocodazole block. One representative of three independent experiments is shown. Costaining of HPV18 E1 and cyclin B1 was carried out 7 days after transfection of U2OS cells with wt HPV18 genomes (G). Two field of views are shown. White arrowheads show HPV replication foci.

Next, the newly synthesized DNA was quantified using primers directed against either HPV or the cellular genome (Fig 3F). Two different pairs of primers were used for the cellular genome, one against early replicating rDNA and the second against the late-replicating Amy gene. The abundance of newly synthesized early replicating cellular DNA was highest at the 9-hour and 12-hour time points in all three cases, while the late-replicating DNA peaked at approximately 15 hours. The replication profile of stably maintained HPV overlapped with that of cellular replication, although the replication profile had a different shape during the initial amplification phase. The signal abundance of both the wt and E8ˉ genomes began to increase at time points corresponding to S phase but peaked at the final time point (20 hours) at which point the majority of cells had reached the G2 phase. No recurrent difference in the replication timing was observed between the HPV18 wt and E8ˉ genomes.

These results are consistent with our previous findings about HPV18 E8ˉ transient replication, that showed the presence of viral replication centers in the cells positive for G2 specific marker cyclin B1 [10]. We next used the same approach to also confirm the continuation of wt HPV18 genome replication in the G2 phase of the cell cycle (Fig 3G). U2OS cells were transfected with wt HPV18 genome and viral replication foci were labeled using anti-E1 antibodies, while cells in G2 were stained using anti-cyclin B1 antibodies. Although expression level of E1 is much lower in case of the wt genome compared to E8ˉ genome, we were able to detect E1 replication foci in some of the cells 7 days after the transfection. Most of the cells containing these foci also had strong cyclin B1 signal in the cytoplasm, indicating that these are the G2 phase cells. Presence of cells in the G2 phase that contain viral genome replication centers is an additional evidence that initial amplification of the wt HPV18 genome can take place during the G2 phase of the cell cycle. Allthogether, results of synchronization and IF experiments suggested that viral genome replication starts in S phase but continues to G2 phase during the initial amplification phase, whereas the HPV genome replicates only in S phase during stable maintenance phase.

### Viral replication in G2-phase is dependent on E1 and E2 protein levels

**Fig 4:**
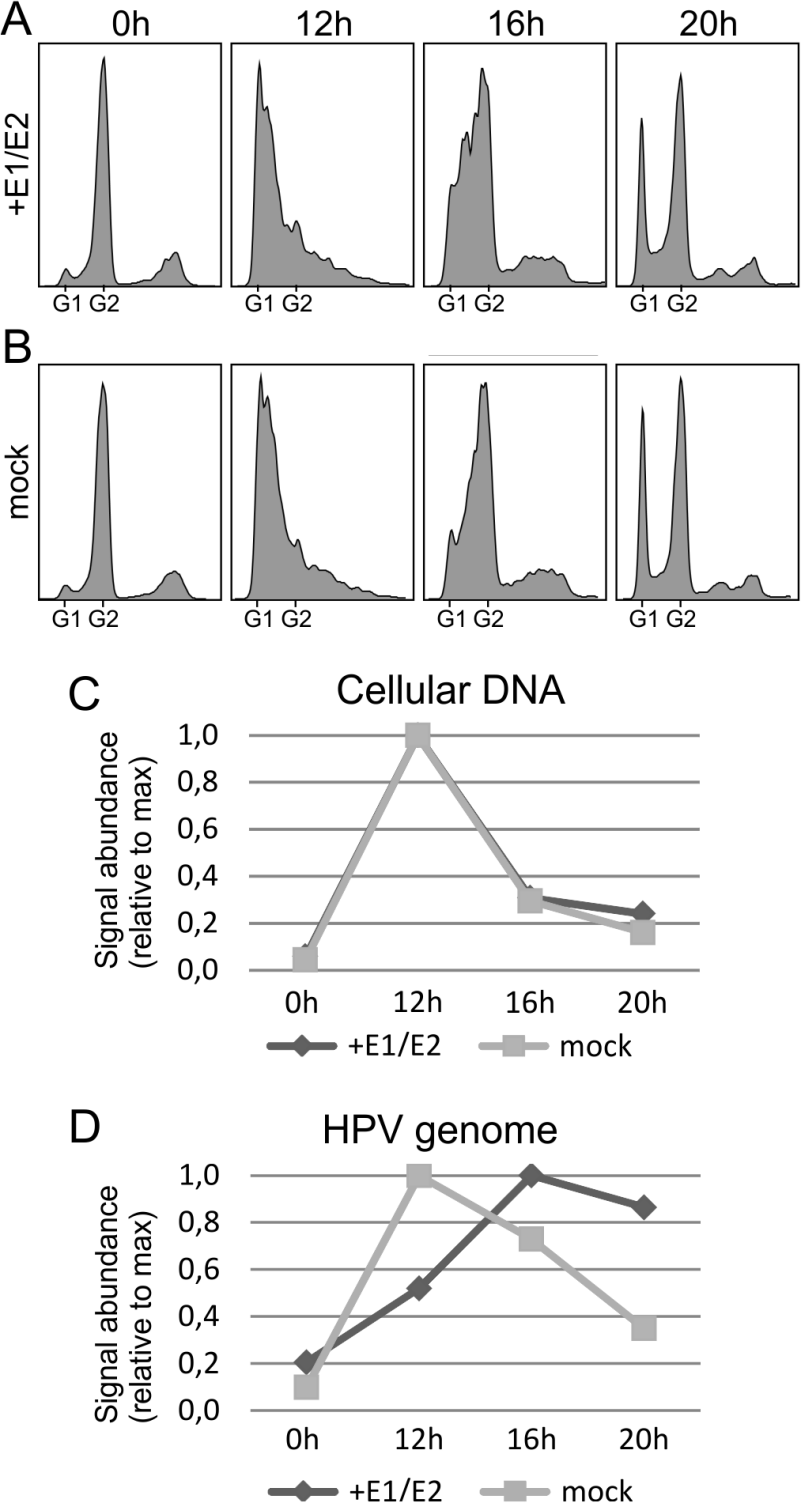
The cell cycle timing of the viral DNA replication is dependent on E1 and E2 levels.

Next, we examined the reason why DNA replication has different cell cycle timing during these first two phases. We hypothesized that the reason may be different expression levels of the viral replication proteins E1 and E2. The E1 and E2 proteins are both necessary for transient HPV amplification, while only extremely low levels of these proteins are present during stable maintenance. A cell cycle synchronization assay similar to the previously described assay was performed using cells that stably maintained HPV18 episomes; however, the cells were transfected with E1 and E2 expression constructs during the synchronization process (Fig 4). Empty vectors not containing the E1 or E2 reading frames were transfected as the mock controls. Because a high level of E1 protein expression is known to cause cell cycle arrest [10, 23], we first ensured that the E1 and E2 levels were low enough in our experiment that they did not interfere with cell cycle progression. The additional expression of E1 and E2 proteins did not cause cell cycle arrest (Fig 4A), and the cell cycle progressed identical to that in the mock transfection (Fig 4B). Next, the newly synthesized cellular DNA was quantified (Fig 4C), and no difference in the replication timing of E1/E2 transfected and mock-transfected samples was observed. However, the replication timing of the viral genome was altered in the presence of higher E1/E2 expression (Fig 4D). In the mock transfection, the newly synthesized viral genome signal peaked during S phase at the 12-hour time point and decreased at later time points. However, the transfection of E1/E2 expression plasmids clearly changed the replication timing profile of viral DNA by moving the signal toward later time points, thus demonstrating that higher E1 and E2 expression allows the HPV genome to replicate during the G2 phase of the cell cycle.

A cell cycle synchronization assay similar to that in Fig 3 was performed on cells that stably maintained HPV18 genome episomes; however, the cells were transfected with either E1 and E2 expression constructs or empty vectors as mock controls during the synchronization process. Cell cycle profiles were determined for E1/E2 (A) and for mock (B) transfected cells. Cellular (C) and viral (D) DNA replication timing was measured, and the signals for newly synthesized DNA abundance were normalized to the maximum value of the series. A representative of two independent experiments is shown.

## Discussion

The present study described the cell cycle timing of HPV18 DNA replication during the first two viral replication phases, namely, initial amplification and stable replication. The U2OS cell line, which was demonstrated previously to be a suitable system for studying the molecular mechanisms of HPV genome replication [19, 22], was used for this analysis. The initial amplification of HPV18 was studied by transiently transfecting U2OS cells with viral genomes as described previously; however, a novel approach was used for stable replication experiments. In our previous work, we subcloned HPV-transfected cells to generate cell lines that stably maintained viral genomes. In the present study, the subcloning step was omitted, and cell pools that stably maintained the HPV18 genome were used instead. The HPV18 genome was maintained as an extrachromosomal episome in these cells for at least several weeks and could still initiate amplification under proper culturing conditions (Fig 1). The viral genome copy number per cell stayed relatively constant during the two-week growth period with normal passaging, meaning that each viral genome was replicated statistically once per cell cycle, which is characteristic of the HPV stable maintenance phase.

We developed a new click chemistry-based assay to quantify the newly synthesized DNA (Fig 2). Analogous approaches that use pulse labeling and purification of newly synthesized DNA with nucleotide analogs have been used previously. Pulse labeling with EdU [24, 25] or biotin-dUTP [26] has been used previously for the isolation of proteins on newly synthesized DNA. Pulse labeling with BrdU, subsequent cell sorting and anti-BrdU immunoprecipitation was used previously to determine the latent replication timing of herpes viruses [27]. Although these protocols differ from that used in the present study, all of these protocols rely on the same principle of purifying DNA synthesized during a precise time window immediately before cell lysis. The quantification of this DNA allows an estimation of how actively certain DNA sequences are replicated in a cell population at a given time. Therefore, this assay differs from traditional assays that are used for viral DNA replication analysis because common qPCR and Southern blot methods measure viral DNA quantity but not the intensity of DNA replication and synthesis. Because the HPV genome copy number is a function of the viral DNA synthesis rate and of the efficiency of its genome segregation, a Southern blot assay cannot fully indicate a change in the viral copy number among different viral mutants. Furthermore, the separation of these processes in the case of HPV is complicated because of the tight linkage between segregation and DNA replication elements in its genome, making the ability to measure one of these processes separately extremely useful. This technique is also clearly useful in cases where the rate of DNA synthesis relative to the DNA copy number changes among different samples, as in the cell cycle synchronization experiment performed in this study or, for example, if the immediate effects of small molecule inhibitors on viral DNA replication are studied. Aphidicolin, which is a known DNA replication inhibitor, is shown as an example of a compound that effectively decreases newly synthesized DNA levels (Fig 2D). Interestingly, although both viral and host DNA replication are extremely sensitive to aphidicolin, some viral DNA continues to be synthesized after 2-hour aphidicolin treatment, while cellular DNA synthesis is completely inhibited. The low level of HPV DNA synthesis in the presence of aphidicolin during both initial amplification and stable maintenance indicates that a polymerase alpha-independent replication mechanism is involved in HPV genome replication. Taken together, these results indicate that this assay may be useful for many areas of HPV research.

Vegetative amplification of the HPV genome has been shown to occur during the G2 phase of the cell cycle [17]. A similar phenomenon was described in our previous work with the HPV18/E8ˉ genome during the initial amplification phase [10]. The present study demonstrated that this phenomenon is also true for the HPV18 wt genome (Fig 3F and 3G), which replicates at a much lower level compared to the E8ˉ mutant genome ([10] and Fig 2C). Both of these genomic variants had similar replication timing (Fig 3F); replication of the viral genome began during S phase but reached its maximum level during G2. In contrast to initial amplification, stable replication of HPV18 occurred exclusively during the S phase of the cell cycle (Fig 3F). This timing is similar to the DNA replication timing of the Epstein-Barr virus (EBV) genome during the latent infection phase [28]. During the latent infection phase, EBV genomes replicate only once per S phase because of the dependence on host cell licensing factors [29]. Both once-per-S-phase and random-choice mechanisms have been described for the stable replication of the HPV genome, depending on the cell line used [30]. The existence of cell lines that support once-per-S-phase HPV genome replication, together with results showing that the E1 protein is dispensable for HPV genome stable replication [31], suggest that viral DNA replication during the stable maintenance phase could be performed solely by host cell replication proteins. Our finding that a difference in DNA replication timing exists between the initial and stable replication stages of HPV genomes is consistent with this hypothesis and indicates that alternative mechanisms are used for genome multiplication during these two phases.

Although DNA viruses are known to use the G2 phase of the cell cycle for their genome replication, the reason for this preference remains unclear. G2-arrested cells may be a good environment for PV DNA replication because no competition with the host cell for DNA synthesis resources exists. However, G2 might not be as suitable as S phase for PV replication because of lower DNA polymerase alpha-primase activity. DNA polymerase alpha-primase activity is regulated by phosphorylation in a cell cycle-dependent manner, and its activity to support SV40 DNA replication begins to decrease after S phase completion [32]. Because polymerase alpha-primase is also an important binding partner of the E1 protein [33], E1-dependent replication may also slow down during G2 phase. Although the change in the cellular environment during G2 could be prevented by expressing the viral oncoproteins E6 and E7, which could establish a so-called pseudo-S phase [34], this possibility does not seem to be the case during the initial amplification phase because E1 and E2 expression alone in the presence of the HPV18 origin is sufficient for establishing viral replication centers in G2 phase cells [1010]. Thus, if the conditions in G2 phase are unfavorable for E1-driven DNA replication, then how does the virus use this phase for its genome replication? One possible explanation could be that the HPV DNA replication mode switches between the S and G2 phases. In addition to E1-driven theta replication, papillomaviruses also use recombination-dependent replication (RDR) for viral genome synthesis. This type of HPV genome replication could occur in G2 phase cells because homologous recombination pathways are active during the G2 phase when sister chromatids are available.

HPV genome replication during the G2 phase seems to be dependent on the E1 protein levels because providing E1 protein to cells that carry stable viral episomes in trans restored replication during the G2 phase (Fig 4). This result is also supported by our previous observation that a high level of expression of the E1 and E2 proteins from expression constructs causes URR-containing plasmid replication during the G2 phase of the cell cycle [1010]. However, if E1-independent RDR is responsible for viral genome synthesis during the G2 phase, then we must ask why viral DNA replication in G2 phase is dependent on the E1 protein. We suggest that E1 and E2 initiate the first round of replication in S phase during the initial amplification phase, creating prerequisites for RDR that can occur during both S and G2 phases. E1, which is a helicase, may be incapable of finishing the replication of circular viral genomes and leave stalled replication forks or other abnormal DNA replication products behind that can be used as substrates for the activation of DNA repair and recombination pathways. Indeed, we demonstrated previously that the ATR pathway, which signals replication stress, is activated at HPV transient replication centers [1010].
